# Recognition of Obstructive Sleep Apnea: An Exploratory Bayesian Modeling Analysis

**DOI:** 10.3390/jpm16050273

**Published:** 2026-05-19

**Authors:** Maria Perifanou-Sotiri, Evaggelia Anyfanti, Eleftherios Meletis, Olympia Lioupi, Chaido Pastaka, Polychronis Kostoulas, Konstantinos I. Gourgoulianis, Garyfallia Perlepe

**Affiliations:** 1 Department of Respiratory Medicine, Faculty of Medicine, School of Health Sciences, University of Thessaly, 41110 Larissa, Greece; cpastaka@gmail.com (C.P.); kgourg@uth.gr (K.I.G.); 2 Faculty of Public and One Health, University of Thessaly, 43100 Karditsa, Greece; eanyfanti@uth.gr (E.A.); elmeletis@uth.gr (E.M.); olioupi@gmail.com (O.L.); pkost@uth.gr (P.K.)

**Keywords:** polysomnography at home, retrospective, comorbidities, AHI, ODI, Bayesian analysis

## Abstract

**Background/Objectives**: Two diagnostic approaches for sleep studies are commonly used worldwide: in-laboratory polysomnography [PSG] and home sleep apnea testing [HSAT]. Although HSAT has gained increasing acceptance due to its convenience and lower cost, clinical criteria for HSAT use remain complex and cannot be inferred directly from AHI/ODI severity indices alone. The aim of the present exploratory study was to examine associations between routinely collected demographic, clinical, and symptom-related variables and objective indices of disease severity, namely the apnea–hypopnea index [AHI] and oxygen desaturation index [ODI] as an initial, hypothesis-generating step toward future patient-level model development and validation. **Methods**: A retrospective observational analysis was conducted in 1100 individuals who previously underwent in lab-polysomnography [PSG] at the University Hospital of Thessaly, Greece, between 2006 and 2023. Specific demographic, clinical and symptom-related variables were included in this study [six continuous and fifteen categorical], which were analyzed in relation to AHI and ODI values. A three-step process was carried out: variable selection followed a screening and backward elimination process. Multivariable linear regression models were subsequently estimated within a Bayesian framework using Hamiltonian Monte Carlo methods. **Results**: Out of 1100 individuals, the mean age was 51.9 years with the predominant gender being male [76%]. Obesity [65.6%] and hypertension [40.5%] were the most common comorbidities. For AHI, male gender, body mass index [BMI], Epworth Sleepiness Scale [ESS] score, reported breathing interruptions during sleep, and chronic obstructive pulmonary disease [COPD] were significant predictors. For ODI, significant predictors included male gender, BMI, ESS score, breathing interruptions during sleep, daytime sleepiness, obesity, and COPD. COPD showed an inverse association with both indices. **Conclusions**: These findings support the feasibility of integrating routinely available clinical variables within a Bayesian probabilistic framework to estimate disease severity pre-test probability. The current analysis may not constitute a validated tool for HSAT versus PSG selection; however, it is an initial, hypothesis-generating step toward future model development.

## 1. Introduction

Obstructive sleep apnea [OSA] is a highly prevalent sleep-related breathing disorder characterized by recurrent episodes of complete [apnea] or partial [hypopnea] upper airway obstruction during sleep, leading to intermittent hypoxemia and sleep fragmentation. It is estimated that nearly one billion individuals worldwide are affected by OSA, making it one of the most common chronic disorders globally [1,2].

Established risk factors for OSA include sex and increasing age. The disorder is more frequently diagnosed in men; however, its prevalence increases significantly in women after menopause, suggesting a potential role of hormonal influences in the pathophysiology of upper airway collapse. Age represents another major determinant, with the mean age of diagnosis typically occurring in middle-aged and older adults [3,4].

Additional well-recognized risk factors include obesity, cardiovascular comorbidities such as hypertension and coronary artery disease and genetic predisposition [5]. OSA is associated with substantial cardiovascular, metabolic, and neurocognitive morbidity, underscoring the importance of timely diagnosis and appropriate patient stratification [6,7].

In addition to the classical risk factors described above, several other mechanisms have been implicated in the pathophysiology of Obstructive Sleep Apnea. These include metabolic conditions such as Type 2 Diabetes, craniofacial skeletal characteristics, upper airway soft tissue abnormalities, and alterations in the position of the hyoid bone. Orthodontic and maxillofacial disorders, such as reduced maxillomandibular volume and posterior rotation of the mandible may also contribute to upper airway narrowing and increased collapsibility during sleep. Furthermore, comorbid insomnia and sleep apnea [COMISA] has been increasingly recognized as a frequent clinical phenotype that complicates diagnosis and management [8].

Certain congenital craniofacial syndromes may further predispose individuals to airway obstruction during sleep. Conditions such as Treacher Collins syndrome and Goldenhar syndrome are characterized by structural abnormalities of the facial skeleton and upper airway that may significantly increase the risk of sleep-disordered breathing [9]. These observations highlight the complex and multifactorial nature of OSA, in which anatomical, physiological, hormonal, and lifestyle-related factors interact to influence disease expression and severity.

Disease severity is conventionally quantified using the apnea–hypopnea index [AHI], defined as the number of apneas and hypopneas per hour of sleep. Hypoxic burden is commonly assessed using the oxygen desaturation index [ODI], which reflects the frequency of oxygen desaturation events. Both indices are typically categorized as mild [5–15 events/h], moderate [15–30 events/h], and severe [>30 events/h] disease [10].

The diagnosis of OSA can be established using either in-laboratory polysomnography [PSG], which remains the gold standard [4], or home sleep apnea testing [HSAT] [11]. Although guidelines for HSAT were first issued by the American Academy of Sleep Medicine [AASM] in 2007, its clinical use has expanded substantially over the past decade. Updated clinical practice recommendations published by the AASM in 2021 further support the use of HSAT in appropriately selected adult patients with suspected OSA [12]. Most HSAT devices assess respiratory events through airflow, respiratory effort, and oxygen saturation monitoring, but do not evaluate sleep architecture due to the absence of electroencephalography [13].

HSAT offers several advantages, including improved patient comfort, reduced waiting times, and lower cost compared to in-laboratory PSG [14]. According to AASM recommendations, HSAT is indicated in patients for whom in-lab PSG is impractical due to immobility, safety concerns, or critical illness, as well as for monitoring non-continuous positive airway pressure [CPAP] treatments such as oral appliances, weight loss, and upper airway surgery. In contrast, HSAT is not recommended for population screening, patients with suspected comorbid sleep disorders [e.g., central sleep apnea, parasomnias, narcolepsy, or periodic limb movement disorder] or individuals with severe comorbidities including neuromuscular disease, congestive heart failure, or prior stroke [15].

In recent years, consumer wearable technologies such as smartwatches and portable monitoring systems have also emerged as potential tools for preliminary detection of sleep-disordered breathing. Although these devices cannot replace formal diagnostic testing, they may contribute to early suspicion of OSA and encourage referral for definitive evaluation [16].

Given these considerations, research that clarifies how routinely collected variables relate to AHI and ODI may inform future efforts to improve diagnostic planning and resource utilization. While previous studies have examined associations between demographic characteristics and indices such as AHI and ODI, fewer studies have attempted to integrate these variables within a probabilistic predictive framework. The primary objective of this study was therefore to conduct an exploratory Bayesian modeling analysis to estimate associations with AHI and ODI and explore pre-test probability. In parallel, we sought to quantify the relationships between AHI, ODI, and key patient characteristics to facilitate early identification of individuals at high risk for clinically significant disease. These results aim to generate hypotheses for future validated decision-support work, including studies that define patient-level thresholds and prospectively evaluate HSAT versus PSG pathways.

## 2. Materials and Methods

### 2.1. Study Population and Data Source

This retrospective study included data from 1100 individuals with suspected sleep-related breathing disorders who underwent diagnostic evaluation at the University Hospital of Larissa, Greece, between 2006 and 2023. The dataset comprised demographic, clinical, and symptom-related variables routinely collected during clinical assessment. The study protocol was reviewed and approved by the Institutional Review Board of the University Hospital of Larissa (protocol code: 22436; approval date: 27 May 2024) and by the Ethics Committee of the Faculty of Medicine, University of Thessaly (protocol code: 622; approval date: 11 June 2024). All procedures were conducted in accordance with the principles of the Declaration of Helsinki and relevant national regulations. The requirement for informed consent was waived due to the retrospective nature of the study.

Eligible participants were adults aged ≥18 years with complete polysomnographic data. Patients with missing demographic or clinical information, incomplete AHI or ODI measurements or poor-quality signal acquisition were excluded from the analysis. Diagnosis and classification of sleep apnea severity were based on established AASM criteria.

AHI and ODI were categorized into four severity groups for descriptive purposes: none [<5 events/h], mild [5–15 events/h], moderate [15–30 events/h], and severe [>30 events/h]. These categorizations are presented in Table 1. For statistical modeling, however, AHI and ODI were analyzed as continuous variables [events/h] to preserve information and maximize statistical power.

### 2.2. Statistical Analysis and Variable Selection

Statistical analyses were performed to evaluate correlations between demographic, clinical, and symptom-related variables with the AHI and ODI. Initially, all independent variables were assessed individually with AHI and ODI as dependent variables. In the first screening phase, variables with *p* < 0.25 were retained for multivariable analysis. In the second stage, backward elimination was applied at a significance level [a] 0.05. Variables with *p* < 0.05 were retained in the final models.

To enhance uncertainty quantification and enable full probabilistic inference, multivariable linear regression models were subsequently estimated within a Bayesian framework as:

$$y=b_{0}+b_{1}x_{1}+b_{2}x_{2}+\cdots +b_{k}x_{k}+e$$

where b_0_ represents the intercept and b the regression coefficients of the predictors X [demographic, clinical and symptom-related variables]. The same specification was used for both dependent variables [AHI and ODI].

The models were implemented using the stan_glm function from the rstanarm package in R [17], with posterior estimation performed using the Hamiltonian Monte Carlo [HMC] algorithm via Stan [18]. Four Markov chains were run, each with 2000 iterations.

Convergence was confirmed through inspection of trace plots and the Gelman–Rubi, n diagnostic [$\hat{R}$ < 1.01 for all parameters]. Low informative priors were used [normal 0, 10]. Variance inflation factors [VIF < 3 for all predictors] indicated no severe multicollinearity. The strong correlation between AHI and ODI supports the modeling strategy of assessing them separately but highlights their clinical interdependence [r = 0.94, *p* < 0.001].

A Bayesian modeling approach was adopted because it allows more informative quantification of uncertainty compared to frequentist estimation, as well as the direct interpretation of credible intervals [CrI], which reflect the probability that a parameter lies within a defined range. The Bayesian framework also allows explicit representation of uncertainty, thereby avoiding rigid classification thresholds and supporting nuanced clinical interpretation.

All analyses were performed in R programming language [19]. Model fit was evaluated using posterior predictive diagnostics and the Bayesian R^2^ statistic, as implemented in the rstanarm package [20]. Model calibration was further assessed by comparing observed data distributions with posterior predictive simulations.

## 3. Results

A total of 1100 participants were included in the analysis. The mean age of the cohort was 51.9 years [95% Confidence Interval [CI]: 51.0–52.7] and the majority were male 76% [95% CI: 72.3–77.9]. The most commonly reported symptoms were snoring 99.4% [95% CI: 98.5–99.7], breathing interruptions in sleep 98.2% [95% CI: 96.7–98.7], and daytime sleepiness 32.3% [95% CI: 29.3–35.4].

Regarding comorbidities, obesity was present in 65.6% [95% CI: 62.7–68.4] of participants, followed by hypertension 40.4% [95% CI: 37.2–43.6], and cardiovascular disease 13.2% [95% CI: 11.3–15.3]. Additional comorbid conditions included diabetes 11% [95% CI: 9.2–13.0], chronic obstructive pulmonary disease [COPD] 6.09% [95% CI: 4.8–7.7], and asthma or rhinitis 7.09% [95% CI: 5.7–8.8]. Baseline demographic and clinical characteristics of the study population are summarized in Table 2.

**Figure 1:**
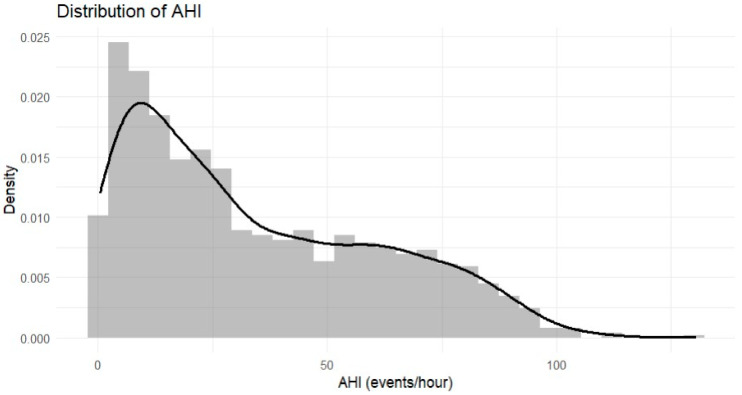
Distribution of the Apnea–Hypopnea Index [AHI] in the study sample.

**Figure 2:**
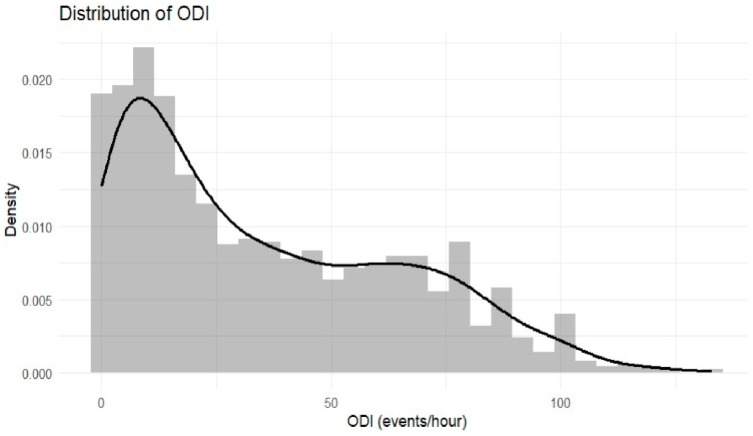
Distribution of the Oxygen Desaturation Index [ODI] in the study sample.

The distributions of AHI and ODI values across the study population are illustrated in Figure 1 and Figure 2, respectively. Both indices demonstrated right-skewed distributions, with a substantial proportion of participants exhibiting moderate to severe disease.

### 3.1. Predictors of AHI and ODI

Associations between demographic, clinical, and symptom-related variables and AHI and ODI were examined using a two-stage modeling approach. In the initial screening phase, variables meeting the predefined inclusion criterion were advanced to multivariable modeling.

For AHI, male gender, Body Mass Index [BMI], Epworth Sleepiness Scale [ESS] score, breathing interruptions during sleep, obesity and COPD were statistically significant predictors [Table 3]. In Bayesian inference, statistical significance is determined by examining whether the 95% credible interval of the posterior distribution excludes zero. The posterior mean estimate for gender was 14.3 [95% CrI: 11.0–17.8], indicating that men had on average 14.3 units higher AHI values compared to women. BMI [estimate: 1.3, 95% CrI 1.0–1.7] and ESS [estimate: 0.7, 95% CrI: 0.3–1.1] were also positively associated with AHI. Chronic obstructive pulmonary disease showed a negative association [estimate: −8.0, 95% CrI −14.0 to −2.0].

For ODI, male gender, BMI, ESS score, reported breathing interruptions during sleep, daytime sleepiness, obesity, and COPD were identified as significant predictors [Table 3]. All predictors, with the exception of COPD, were positively associated with ODI values. Male participants exhibited higher ODI values than female participants [estimate: 13.2; 95% CrI: 9.7–16.8]. BMI showed a strong positive association with ODI [estimate: 1.8; 95% CrI: 1.4–2.1]. Daytime sleepiness was significantly associated with ODI but not with AHI.

### 3.2. Model Fit and Calibration

Posterior predictive diagnostics indicated good model calibration for both AHI and ODI models, with predicted values closely matching the observed data [Table 2]. The final models demonstrated moderate explanatory power, with Bayesian R^2^ values of 0.25 [95% CrI: 0.21–0.29] for the AHI model and 0.29 [95% CrI: 0.25–0.33] for the ODI model. These values indicate that approximately 25% of the variability in AHI and 29% of the variability in ODI are explained by the model’s predictive factors. Although modest, such levels of explained variance are typical for clinical observational data [21].

## 4. Discussion

The present exploratory study examined associations between routinely demographic, clinical and symptom-related variables and objective indices of disease severity, namely AHI and ODI, in individuals with suspected sleep-related breathing disorders. The main findings indicate that male gender, increased BMI, higher ESS scores, and reported breathing interruptions during sleep are associated with greater severity of sleep-disordered breathing, as reflected by both AHI and ODI. In contrast, COPD exhibited an inverse association with both indices, while daytime sleepiness was significantly associated with ODI but not AHI.

These associations were consistent across both frequentist screening and Bayesian multivariable modeling approaches, supporting the robustness of the identified predictors. The Bayesian framework further enabled probabilistic interpretation of effect estimates and uncertainty, which is particularly relevant in observational clinical data. Importantly, the aim of the present study was not simply to confirm previously described associations, but to integrate routinely collected clinical variables within a Bayesian probabilistic framework to support estimation of pre-test probability estimation for clinically significant sleep-disordered breathing and inform future HSAT/PSG pathway research.

The strong association between male gender and higher AHI and ODI observed in this study is well established in the literature. Anatomical and hormonal differences, including upper airway morphology and fat distribution, contribute to increased airway collapsibility in men, resulting in higher disease severity and greater hypoxic burden [4,22,23,24]. Similarly, obesity remains one of the most powerful predictors of obstructive sleep apnea severity. Increased BMI is associated with fat deposition in the upper airway and reduced lung volumes, both of which exacerbate airway obstruction during sleep. Prior studies have demonstrated that even modest increases in body weight are associated with measurable increases in AHI, a finding that aligns closely with the present results [4].

Higher ESS scores were positively associated with both AHI and ODI, indicating that subjective daytime sleepiness reflects underlying sleep fragmentation and nocturnal hypoxemia. However, reported daytime sleepiness was significantly associated only with ODI and not with AHI. This finding supports previous evidence suggesting that AHI alone is an imperfect surrogate for symptomatic burden and that measures of oxygen desaturation may better capture the physiological mechanisms contributing to excessive daytime sleepiness [25,26,27,28,29,30]. These observations further reinforce the clinical relevance of incorporating ODI, in addition to AHI, when assessing disease severity and patient impact.

Reported breathing interruptions during sleep emerged as a strong predictor of both AHI and ODI, consistent with its role as a cardinal symptom of obstructive sleep apnea. This finding supports the inclusion of witnessed apneas as a key variable in future screening or prediction models that estimate the pre-test probability of clinically significant sleep-disorder breathing.

The prevalence of rhinitis in our cohort appeared lower than that reported in the literature, where approximately 10–20% of the general population is affected [31]. Both allergic and non-allergic rhinitis have been associated with impaired sleep quality and the development of sleep-related breathing disorders, including Obstructive Sleep Apnea, primarily through nasal obstruction, increased upper airway resistance, and mouth breathing [4]. The increasing prevalence of rhinitis over recent decades has been linked to environmental and lifestyle factors, such as urbanization, air pollution, reduced microbial exposure, and climate-related changes affecting allergen levels [32,33,34]. The lower prevalence observed in our study may reflect underreporting or differences in study design. Nevertheless, rhinitis remains clinically relevant due to its potential contribution to the pathophysiology of OSA.

The inverse association between COPD and both AHI and ODI may appear counterintuitive given the recognized coexistence of obstructive sleep apnea and COPD in overlap syndrome and should be interpreted with caution. COPD was present in only approximately 6% of the cohort and was recorded as a binary comorbidity. Residual confounding and referral bias may therefore have influenced the estimate. This result should not be interpreted as evidence that COPD reduces the risk of clinically important sleep-disordered breathing. Clinically significant COPD or suspected hypoventilation remains an important contextual factor for future HSAT/PSG pathway research, because simplified home testing may not adequately characterize complex respiratory physiology. However, several studies have reported lower AHI values among COPD-predominant patients, likely reflecting differing pathophysiological mechanisms of nocturnal hypoxemia. In COPD, sustained hypoventilation and ventilation–perfusion mismatch may predominate over discrete obstructive respiratory events, resulting in lower AHI despite clinically relevant oxygen desaturation patterns [35,36,37,38,39]. These findings highlight why pulmonary comorbidity should be considered carefully in future validation studies of HSAT/PSG pathway models, because in-laboratory polysomnography may better characterize complex respiratory and gas exchange abnormalities in such patients.

Beyond the predictors examined in the present model, another aspect that has received increasing attention in recent years is the potential relationship between temporomandibular disorders (TMDs) and Obstructive Sleep Apnea. Craniofacial morphology, occlusal characteristics, and mandibular positioning may influence upper airway patency during sleep and have therefore been implicated in the pathophysiology of sleep-disordered breathing [40]. In particular, reduced maxillomandibular volume and posterior rotation of the mandible have been associated with decreased upper airway dimensions and increased airway collapsibility during sleep. Several studies have reported a higher prevalence of temporomandibular symptoms among patients with OSA, potentially related to increased masticatory muscle activity, sleep bruxism, or biomechanical alterations of the craniofacial complex. In addition, therapeutic interventions for OSA, such as mandibular advancement devices, directly modify mandibular position and may influence temporomandibular joint loading. Although the causal relationship between TMDs and OSA remains incompletely understood, these findings highlight the importance of interdisciplinary collaboration between sleep physicians, dentists, and maxillofacial specialists when evaluating patients with suspected sleep-disordered breathing [8].

From a clinical perspective, the present findings have implications for future diagnostic pathway studies in sleep medicine. The consistent association of readily obtainable variables—such as gender, BMI, ESS score, breathing interruptions during sleep, and daytime sleepiness—with AHI and ODI suggests that these features may help estimate pre-test probability of clinically significant sleep-disordered breathing in future validated models. Integrating these predictors into a structured, algorithm-based triage system may improve identification of patients most likely to benefit from HSAT, while reducing unnecessary in-laboratory studies in lower-risk or more complex cases [41].

Such an approach aligns with increasing pressures on sleep laboratories, where access to in-lab polysomnography is often limited by cost, personnel, and waiting times. A data-driven triage strategy could prioritize high-probability patients for HSAT, facilitate earlier diagnosis and treatment initiation, and allocate laboratory-based resources to patients with atypical physiology or significant comorbidities.

Beyond its clinical utility, future development of validated probabilistic decision-support tools in sleep medicine raises important ethical considerations related to fairness, proportionality, and responsible stewardship of limited healthcare resources. In the context of obstructive sleep apnea, where diagnostic demand frequently exceeds the capacity of sleep laboratories, clinical choices regarding in-laboratory polysomnography versus home sleep apnea testing inevitably may involve implicit prioritization. When such decisions remain unstructured, they may be influenced by subjective judgment, local availability, or non-clinical factors. The explanatory approach evaluated here aligns with core principles of biomedical ethics, particularly beneficence and justice [42], by integrating routinely collected clinical and symptom-related variables into a transparent probabilistic framework. Future steps involve development of a tool that might help clinicians interpret pre-test probability, while ensuring that individuals with complex comorbidities, such as chronic obstructive pulmonary disease, remain assessed according to established clinical criteria rather than model output alone. Importantly, the Bayesian framework offers ethical advantages over rigid deterministic rules: rather than imposing fixed classifications, it explicitly represents uncertainty and allows risk to be interpreted along a continuum. This emphasis on transparency and interpretability is increasingly recognized as central to the responsible integration of model-based systems in clinical medicine [43,44]. Such transparency preserves clinical judgment, supports shared decision-making, and positions models as decision-support aids rather than decision-making authorities [45]. Nevertheless, the ethical legitimacy of such model-based approaches depends on careful external validation, monitoring for potential bias, and evaluation of patient-centered outcomes, consistent with emerging international guidance on AI governance in health [46]. The present findings should therefore be viewed as an exploratory hypothesis-generating step toward clinically and ethically informed model development, not as a definitive rule for access to care. Before any clinical implementation, the model would need to be converted into a patient-level prediction tool and evaluated using internal validation, external validation, calibration assessment, and clinically meaningful decision thresholds. Candidate thresholds should be selected according to the intended use of the model, for example prioritizing sensitivity when the goal is to avoid missing moderate-to-severe OSA or prioritizing specificity when the goal is to preserve PSG capacity for complex cases.

Overall, the integration of routinely collected clinical variables within a Bayesian probabilistic framework may provide a transparent exploratory approach for estimating pre-test probability in suspected obstructive sleep apnea and informing the future development of validated HSAT/PSG decision-support models.

Several limitations should be acknowledged. First, the current model does not define thresholds or decision rules for HSAT eligibility. Second, the present analysis is based on an interim dataset, and data collection is ongoing. As the sample size increases, effect estimates—particularly for less prevalent comorbidities—may change. Consequently, the reproducibility of the posterior estimates and the transportability of the model to other healthcare settings, referral patterns, ethnic backgrounds, HSAT devices, and scoring practices are unknown. Third, although internal model performance was acceptable, external validation has not yet been performed, and generalizability to other populations and clinical settings remains to be established. Variables such as hypoxic burden and T90% were not available in the present dataset and therefore could not be included in the analysis. Future studies should investigate whether incorporating these parameters could further improve predictive models for HSAT indication. Finally, while the modeling framework is intended to inform the future development of a clinical decision-support model, prospective implementation and evaluation of its impact on diagnostic efficiency and patient outcomes have not yet been assessed. Moreover, algorithm-based triage systems require prospective validation and monitoring to ensure that implementation does not introduce unintended bias or reduce individualized clinical assessment.

## 5. Conclusions

In this retrospective analysis, male gender and obesity emerged as the strongest predictors of increased AHI and ODI, while ESS score and reported breathing interruptions during sleep were also consistently associated with greater disease severity. In contrast, COPD demonstrated an inverse relationship with both indices, and daytime sleepiness was associated with ODI but not AHI, underscoring the limitations of AHI alone as a marker of symptomatic burden.

These findings support the use of routinely collected demographic, clinical, and symptom-related variables to estimate pre-test probability of clinically significant sleep-disordered breathing. Incorporating such variables into a future structured, algorithm-based triage approach may evaluate HSAT/PSG diagnostic pathways, optimize diagnostic yield, and support more efficient allocation of sleep laboratory resources. However, the current analysis does not provide decision rules for patient selection. Future work will focus on expanding the dataset, externally validating the proposed model, defining clinically thresholds and assessing its clinical utility in real-world HSAT diagnostic pathways.

## Figures and Tables

**Table 1:** Population characteristics [demographic, clinical and symptom-related variables] of Apnea–Hypopnea Index [AHI] and Oxygen Desaturation Index [ODI] severity groups.

		AHI Group					ODI Group				
Variables		None * n [%]	Mild n [%]	Moderate n [%]	Severe n [%]	Total [100%]	None n [%]	Mild n [%]	Moderate n [%]	Severe n [%]	Total [100%]
Gender	Male	61 [7.3%]	164 [19.6%]	185 [22.2%]	426 [50.9%]	836	89 [10.6%]	146 [17.5%]	159 [19.0%]	442 [52.9%]	836
	Female	63 [23.9%]	68 [25.7%]	56 [21.2%]	77 [29.2%]	264	63 [23.8%]	67 [25.4%]	48 [18.2%]	86 [32.6%]	264
Snoring		122 [11.2%]	230 [21.0%]	240 [21.9%]	502 [45.9%]	1094	150 [13.7%]	211 [19.3%]	207 [18.9%]	526 [48.1%]	1094
Breathing interruptions in sleep		117 [10.8%]	225 [20.8%]	239 [22.1%]	500 [46.3%]	1081	145 [13.4%]	210 [19.4%]	203 [18.8%]	523 [48.4%]	1081
Daytime sleepiness		27 [7.6%]	61 [17.1%]	71 [19.9%]	197 [55.4%]	356	35 [9.8%]	55 [15.4%]	51 [14.4%]	215 [60.4%]	356
Hypertension		31 [7.0%]	71 [16.0%]	99 [22.2%]	224 [54.8%]	425	38 [8.5%]	62 [13.9%]	87 [19.6%]	258 [58%]	445
Cardiovascular disease		10 [6.9%]	24 [16.5%]	31 [21.4]	80 [55.2%]	145	14 [9.6%]	23 [15.9%]	27 [18.6%]	81 [55.9%]	145
Diabetes		10 [8.2%]	14 [11.6%]	25 [20.7%]	72 [59.5%]	121	11 [9.1%]	15 [12.4%]	15 [12.4%]	80 [66.1%]	121
Obesity		55 [7.6%]	114 [15.8%]	138 [19.1%]	415 [57.5%]	722	48 [6.6%]	111 [15.4%]	123 [17.1%]	440 [60.9%]	722
COPD		5 [7.5%]	9 [13.4%]	17 [25.4%]	36 [53.7%]	67	6 [8.8%]	8 [11.8%]	14 [20.6%]	40 [58.8%]	68
Asthma—Rhinitis		11 [14.1%]	19 [24.3%]	18 [23.1%]	30 [38.5%]	78	11 [14.1%]	20 [25.6%]	11 [14.1%]	36 [46.2%]	78
Free		41 [21.6%]	64 [33.7%]	51 [26.8%]	34 [17.9%]	190	63 [33.2%]	56 [29.5%]	36 [18.9%]	35 [18.4%]	190
Other background		41 [9.6%]	79 [18.4%]	95 [22.1%]	214 [49.9%]	429	49 [11.5%]	81 [18.9%]	77 [17.9%]	222 [51.7%]	429

* None = [<5 events per h], Mild = [5–15 events per h], Moderate = [15–30 events per h], and Severe = [>30 events per h].

**Table 2:** Baseline sociodemographic and clinical characteristics of the study participants.

Characteristics	Mean ± SD * or n [%]
Age	51.9 ± 12.9
BMI	32.3 ± 5.8
ESS	9.3 ± 4.2
AHI	34.2 ± 27
ODI	35.6 ± 29.4
Gender	Male: 836 [76%]
	Female: 264 [24%]
Symptoms	
Snoring	1094 [99.4%]
Breathing interruptions in sleep	1081 [98.3%]
Daytime sleepiness	356 [32.4%]
Comorbidities	
Hypertension	445 [40.5%]
Cardiovascular disease	145 [13.2%]
Diabetes	121 [11%]
Obesity	722 [65.6%]
COPD	67 [6.1%]
Asthma—Rhinitis	78 [7.1%]
No medical history	190 [17.3%]
Other	429 [39%]

* SD = standard deviation.

**Table 3:** Posterior estimates [posterior means, 95% credible intervals, and standard deviations] from Bayesian linear regression models for AHI and ODI.

	AHI		ODI	
Variables	Estimate [Mean]	95% CrI * [2.5%, 97.5%]	Estimate [Mean]	95% CrI * [2.5%, 97.5%]
Gender [male]	14.3	[11.0, 17.8]	13.2	[9.7, 16.8]
BMI	1.3	[1.0, 1.7]	1.8	[1.4, 2.1]
ESS	0.7	[0.3, 1.1]	0.5	[0.1, 1.0]
Breathing interruptions in sleep	12.7	[3.5, 21.8]	10.1	[0.6, 19.4]
Daytime sleepiness	-	-	3.9	[0.7, 7.8]
Obesity	4.4	[0.1, 8.8]	5.2	[0.3, 9.8]
COPD	−8.0	[−14.0, −2.0]	−5.2	[−11.5, −0.8]
Free	−6.2	[−11.1, −1.1]	−7.4	[−13.0, −1.8]

* CrI = Credible interval.

## Data Availability

The data presented in this study are available upon reasonable request from the corresponding author.

## Abbreviations

AASM	American Academy of Sleep Medicine
AHI	Apnea–Hypopnea Index
BMI	Body Mass Index
CI	Confidence Interval
COPD	Chronic Obstructive Pulmonary Disease
CPAP	Continuous Positive Airway Pressure
CrI	Credible Interval
ESS	Epworth Sleepiness Scale
HMC	Hamiltonian Monte Carlo
HSAT	Home Sleep Apnea Testing
ODI	Oxygen Desaturation Index
OSA	Obstructive Sleep Apnea
PSG	Polysomnography
